# Linking behavioural type with cannibalism in Eurasian perch

**DOI:** 10.1371/journal.pone.0260938

**Published:** 2021-12-03

**Authors:** Matilda L. Andersson, Kaj Hulthén, Charlie Blake, Christer Brönmark, P. Anders Nilsson

**Affiliations:** 1 Division of Aquatic Ecology, Department of Biology, Lund University, Lund, Sweden; 2 Department of Environmental and Life Sciences—Biology, Karlstad University, Karlstad, Sweden; University of Florida, UNITED STATES

## Abstract

The propensity to kill and consume conspecifics (cannibalism) varies greatly between and within species, but the underlying mechanisms behind this variation remain poorly understood. A rich literature has documented that consistent behavioural variation is ubiquitous across the animal kingdom. Such inter-individual behavioural differences, sometimes referred to as personality traits, may have far-reaching ecological consequences. However, the link between predator personality traits and the propensity to engage in cannibalistic interactions remains understudied. Here, we first quantified personality in Eurasian perch (*Perca fluviatilis*), measured as activity (time spent moving) and sociability (time spent near conspecifics). We then gave perch of contrasting behavioural types the option to consume either conspecific or heterospecific (roach, *Rutilus rutilus*) prey. Individual perch characterized by a social-active behavioural phenotype (n = 5) selected roach before being cannibalistic, while asocial-inactive perch (n = 17) consumed conspecific and heterospecific prey evenly. Thus, asocial-inactive perch expressed significantly higher rates of cannibalism as compared to social-active individuals. Individual variation in cannibalism, linked to behavioural type, adds important mechanistic understanding to complex population and community dynamics, and also provides insight into the diversity and maintenance of animal personality.

## Introduction

Cannibalism, the act of killing and consuming conspecifics (intraspecific predation), is widespread in nature. It has been documented in a diverse array of animals and likely occurs in virtually all major vertebrate and invertebrate groups, though the propensity for cannibalism varies between species [1, 2]. From being historically regarded as atypical behaviour or noise, we now know that cannibalism is heritable, has fitness-related consequences, and thus is subject to evolutionary processes [3, 4]. Furthermore, cannibalism can influence a variety of direct and indirect interactions among individuals and drive variation in the density of animals, which can have profound effects upon population, community, and ecosystem dynamics via size-structured cannibal-prey interactions and feedbacks on population and community compositions [5–8]. In addition to variation in cannibalism between populations, variability in the tendency to engage in cannibalistic behaviours may also occur between individuals within populations [9–12]. Cannibalism is particularly well documented in fish populations, and fishes are thus excellent experimental models to study the causes and consequences of intraspecific predation [2, 13]. This behaviour is also of particular interest in the context of fisheries because of its effect on individual fitness and total biomass of cultured species [14, 15]. Cannibalism in fishes frequently occurs as sibling, egg, or filial cannibalism, but also between adults and unrelated individuals [13]. It can act to regulate population density and size structure, give early hatching individuals a competitive edge, or even function as a “lifeboat mechanism” for larger individuals, replacing other food sources during times of prey shortage [16, 17]. However, which mechanisms underlie these inter-individual differences in cannibalistic behaviours still remain unclear.

Evidence from a continuously growing body of literature suggests that individuals from the same population often show consistent differences in a broad spectrum of behavioural traits, including activity, sociability, and risk-taking propensity [18–20]. Correlations between these inter-individual differences in key behavioural traits, over time and across contexts, are commonly referred to as a behavioural syndrome, and within a syndrome, individuals can be classified into different behavioural types such as active vs. inactive [21]. Contemporary research implies that personality variation may have far-reaching implications for evolutionary and ecological processes [22–26]. For example, predator behavioural type has been shown to influence foraging efficiency and the type of prey a given predator kills and consumes [27, 28]. We are, however, only beginning to understand how the behavioural types of predators shape the complex interactions with their prey.

In this study, we aim to examine the link between behavioural type and the propensity for cannibalism in Eurasian perch (*Perca fluviatilis*, Linnaeus 1758, Percidae). Perch are important predators in temperate lakes and are commonly involved in size-structured predator-prey interactions and cannibalism, adding to the complex interactions in lake communities [29, 30]. Perch have also been evaluated for different aspects of animal personality, including risk taking (in groups and alone), activity, and sociability [31–33]. Here, we first quantified two key behavioural traits (sociability and activity) in wild-caught perch, and based on the outcome of these assays, we statistically classified fish into two behavioural phenotypes (social-active or asocial-inactive). We then conducted prey-choice experiments where we presented focal perch of the two behavioural types with the option to consume con- or heterospecifics, under the hypothesis that perch behavioural type influences cannibalism propensity.

## Materials and methods

### Focal fish

Age 1+ perch, standard length (SL): range 75–105 mm, N = 53, were collected by electrofishing in the lake Krankesjön, Sweden (55°42’N, 13°28’E, electrofishing permit from the County Administrative Board of Skåne, fishing permit from owner Revingeby Samfällighet), in August 2015. Fish were then transported to laboratory facilities at Lund University, where they were sorted according to body size and housed in two separate holding tanks (18°C, 250 L). Perch were fed de-frosted red chironomid larvae daily, at a rate corresponding to 15% of total perch wet weight. After two weeks of acclimatization to the laboratory environment, focal perch were anaesthetized (Benzocaine) and individually tagged by surgically implanting a passive integrated transponder tag (PIT-tag; Texas Instruments, TRPGP40TGC; half duplex, 134.6 kHz, 12 mm long, 2.12 mm diameter, 0.10 g in air; www.ti.com) into the stomach cavity via a 0.25 mm incision posterior to the left pelvic fin [34]. Fish were then given two weeks to recover before trials were initiated on all 53 individuals.

### Behavioural assays

Over a two-week period, each of the 53 focal perch was assayed twice for both activity (time spent moving) and sociability (time spent close to a stimulus shoal of conspecifics). The behavioural assay arena consisted of an 80 L circular acrylic tank (diameter 63 cm, height 25 cm), filled to a depth of 10 cm with aged and aerated tap water (18°C). In the centre of the arena, a 4.5 L clear acrylic cylinder (diameter 14 cm) held a stimulus shoal made up of 3 perch individuals (originating from lake Krankesjön, caught at the same time as focal individuals), novel to and of similar size to the focal perch. The clear acrylic cylinder allowed visual, but not physical or olfactory, interaction between the stimulus shoal and the focal perch. Stimulus shoal fish were introduced 5 min before the focal perch, which were then acclimatized for 20 min before the 10 min recording. Recordings of behavioural trials were taken from a top-down view (Logitech HD pro C920 webcam linked to a computer running Evocam v.4.3) at a rate of 30 frames per second. The earliest trials began at 8:55, and the latest trials began at 19:42, and four trials were recorded simultaneously. To assess general activity and sociability, we measured movement and spatial use of the focal perch in the presence of the novel conspecifics. Activity was quantified from the videos as the amount of time (s) spent moving (start speed 2 cm/s, stop 1.75 cm/s), while sociability was quantified as the amount of time (s) a fish spent with its centre of gravity within 8 cm (*c*. one body length) of the stimulus shoal. Both behaviours were analysed at a rate of 5 samples/s using automated tracking software (Noldus Ethovision v.3.1). The average time spent moving and time spent within 8 cm of the stimulus shoal were calculated as the average of trials 1 and 2, and these averages were used in subsequent analyses. Focal individuals were starved for 24 h before each assay in order to standardize hunger levels and given a minimum of 3 days off between the first and second trial. Both activity and sociability behaviours were evaluated during the same trial in an attempt to reduce handling and stress of the experimental subjects. Half of the water was replaced between each trial, and all subjects were assayed twice with a mean of 5.8 days (range 3–9 days) between the first and second trial. Individual scores for activity and sociability were used to classify individuals into behavioural types.

### Classification into behavioural types

The presence of a behavioural syndrome in the 53 perch was evaluated with a correlation analysis between activity and sociability using Spearman’s rank correlation, where ρ > 0.3 and p < 0.05 suggest the presence of a behavioural syndrome, i.e., suites of correlated behaviours across situations [35, 36]. A single-linkage cluster analysis on activity and sociability scores was then used evaluate whether the perch individuals grouped into behavioural types. The difference between the obtained clusters was confirmed using a discriminant function analysis on activity and sociability scores. The 53 assayed perch individuals were evaluated for behavioural consistency between their two activity scores by analysis of their coefficients of variation (CV, $\sigma /\bar{x}$). The 22 fish with the lowest activity CV were selected as experimental individuals resulting in two groups with distinct behavioural types (5 individuals characterized social-active, 17 individuals characterized asocial-inactive). These 22 fish were evaluated for differences between behavioural-type groups regarding body condition (Fulton’s K = M/SL^3^, M = body mass (g) and SL = standard length (cm) [37]) using Wilcoxon rank sum tests.

### Prey fish

Roach and perch (size range 46–57 mm SL) were collected by sink netting in the lake Ringsjön, Sweden (55°52’N, 13°30’E, fishing permit from owner Ringsjöfisk), in November and December 2015. Prey fishes were collected from a different lake than the focal perch to prevent possible kin discrimination from influencing prey selection [38–40]. Prey fishes were maintained in mixed-species shoals in two holding tanks (temp 18°C, volume 250 L tap water) and acclimatized to lab conditions for 24 h before use in predation trials. Prey fish were fed, to satiation, daily with de-frosted red chironomids and *Daphnia pulex*.

### Selective cannibalism

The 22 experimental perch were placed individually in 55 L aquaria filled with 18°C aerated tap water to a depth of 22 cm. Each tank was covered with white vinyl on three sides, had gravel on the bottom, and half of each tank had artificial vegetation (2500 green strings m^-2^) to add structure according to fish welfare regulations. Experimental predatory perch were starved for 48 h to standardize hunger levels, after which 5 prey perch (52.25 ± 2.43 mm SL) and 5 prey roach (51.4 ± 2.43 mm SL) prey were measured, size-matched (max difference 8 mm), and then added simultaneously to the open area of each experimental tank. Tanks were checked every 24 h, and the surviving prey fish were recorded and fed with de-frosted red chironomids at a rate of 20% of the original prey weight. Trials stopped when each focal perch had consumed 3 prey fish, with no trial exceeding 5 days. The remaining prey fishes were not re-used in later trials. Individual predator selectivity between prey types was calculated using the Manly-Chesson selectivity index (*α*) for a decreasing population: $\alpha_{i}=ln[(n_{i0}-r_{i})/n_{i0}]/\sum_{j=1}^{m}ln[{(n}_{j0}-r_{j})/n_{j0}],\;i=1,2,\ldots .m$ [41], since prey was not replaced after each predation event, meaning the ratio of prey changed over the course of the trial. The difference between observed (*α*) and random (*α*_0_ = 0.5) selectivity was evaluated with one-sample Wilcoxon signed-rank tests (V) using the *α* for perch prey for the two behavioural types, respectively. Moreover, differences in cannibalism between the two behavioural types were calculated using an independent Wilcoxon rank sum test (W) on the respective *α* values. We also fit simple linear models on Manly-Chesson selectively index using average movement and average shoaling as explanatory variables. All statistical analyses were performed in R Studio [42]. All experiments were performed under evaluation and permission from the Malmö/Lund authority for ethics of animal experimentation (licence M36-14).

## Results

Average time spent shoaling and average activity across the 53 individual perch were positively correlated (ρ = 0.56, p < 0.001). The cluster analysis grouped the focal individuals into two distinct groups based on their activity and sociability scores, and the two groups were confirmed by discriminant analysis (λ = 0.339, p < 0.001). There were no differences in body condition (Fulton’s K) or body size (SL) between the two behavioural-type groups from the 22 experiment individuals (Wilcoxon rank sum test: W = 26.5, p > 0.5, W = 48.5, p > 0.1, respectively).

**Fig 1:**
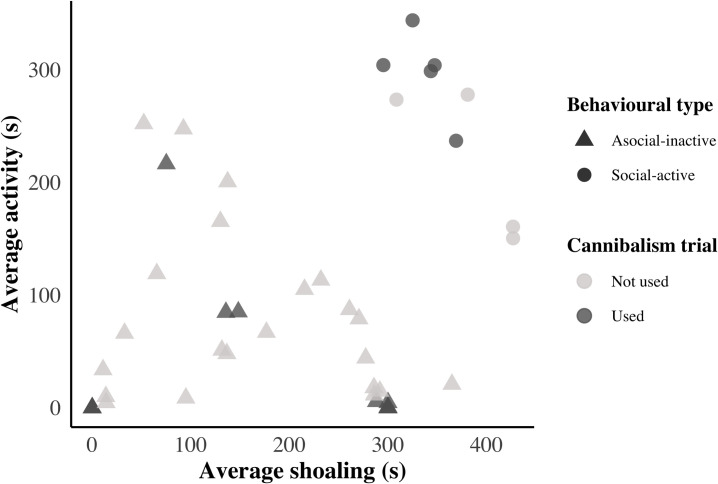
Mean swimming activity (s) in relation to mean shoaling (s) for all tested perch (N = 53). Circles represent the fish classified (cluster analysis) as social-active behavioural type (N = 9), while triangles represent asocial-inactive fish (N = 44). Dark symbols denote the 22 individuals selected (based on CV, $\sigma /\bar{x}$) as experimental fish for the selective cannibalism trials. Note that multiple fish had 0 activity and 0 sociability meaning the triangle located closest to the origin represents multiple individuals.

**Fig 2:**
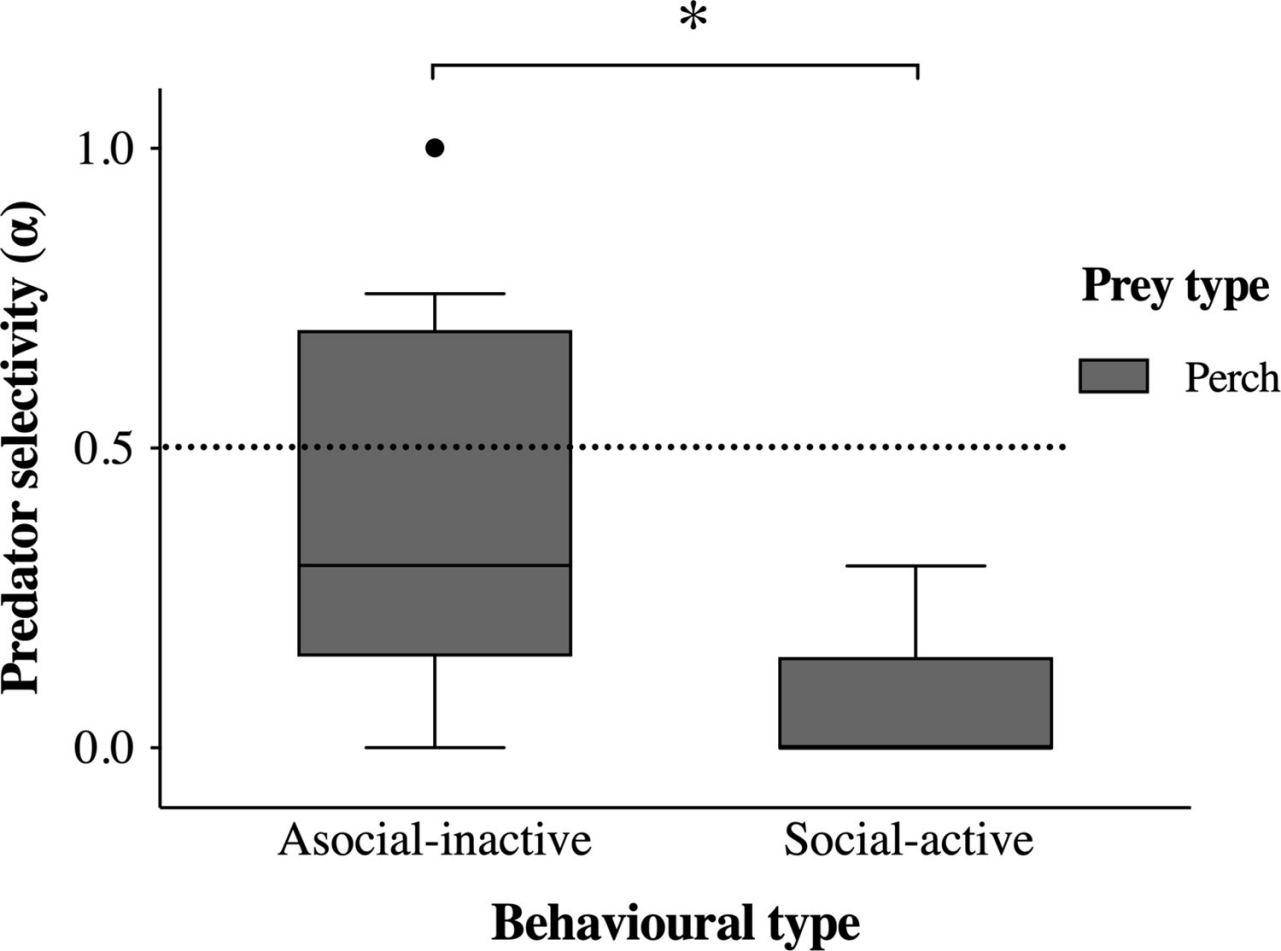
Selection of prey perch by focal perch with social-active (n = 5) and asocial-inactive (n = 17) activity/sociability behavioural types during selective cannibalism trials. Whiskers represent 10^th^– 90^th^ percentile, filled dots represent outliers, and the broken line denotes no prey selectivity (α = 0.5). The * denotes a significant difference at the 0.05 > p > 0.01 level.

Social-active individuals (N = 5) and asocial-inactive (N = 17) behavioural types (Fig 1) differed significantly in their selective cannibalism (W = 69.5, p = 0.025, Fig 2). Perch of the social-active behavioural type selected roach before conspecifics at a rate significantly different from random (V = 0, p = 0.048, Fig 2), while the asocial-inactive behavioural type perch did not differ from random selectivity (V = 47.5, p = 0.158, Fig 2). Linear models showed that neither average movement or average shoaling alone predicted individual propensity for cannibalism (R^2^ = 0.076, p = 0.115, and R^2^ = 0.019, p = 0.248, respectively).

## Discussion

Our experiment reveals a novel link between behavioural types and cannibalism rates among predatory perch. We found that perch characterized by relatively low levels of activity and sociability were more cannibalistic than individuals characterized by relatively high activity and sociability. Linking phenotypic variation to the propensity for cannibalism in predator populations should enhance our understanding of causes and consequences of cannibalism, and thereby our understanding of higher-order ecological processes [6]. The activity levels of individual predators can be linked to foraging strategies [43, 44] and it is possible that our fish with contrasting behavioural types used different strategies and thereby consumed different prey species. Small perch use complex habitats to hide from predators while roach use evasive jumps and comparatively high swimming speeds to evade predation [45]. The difference in predator avoidance strategies of perch and roach may impact the likelihood of encountering perch with different behavioural types or the outcome of the encounters.

We found that the social-active perch phenotype was less cannibalistic and selectively preyed on the more actively swimming roach. In contrast, the asocial-inactive perch phenotype showed no significant selection for either prey species, resulting in a comparatively higher rate of cannibalism. Interestingly, this is contrary to the locomotor crossover hypothesis, which predicts that less active prey are consumed by more active predators and *vice versa* [46]. It also suggests that while perch are efficient cannibals, as is true for many piscivores, there is intraspecific variation in the propensity for this feeding behaviour [47]. Higher consumption of roach by social-active individuals could also be linked to a higher pace of life [48], including higher metabolic rates and attack efficiency [49]. The high activity rates, metabolic rates, and active foraging strategies associated with a fast pace-of-life are advantageous in structurally simple habitats where roach are most often found [48, 50, 51]. Previous research points towards species-specific antipredator behavior [52] in which roach demonstrate comparatively higher swimming speeds and form more dense schools, relative to perch [52, 53]. As roach preferentially use these tactics in open water environments, the social-active perch predator phenotype may be competitively superior at capturing roach individuals. Perch have been shown to preferentially forage in groups and are more efficient when they do so [54]. This may help to explain why it is behavioural type (social-active vs. asocial-inactive) and not activity alone that corresponds with the consumption of heterospecific prey.

Cannibalism may, regardless, have a substantial influence on community and systems composition [6, 47, 55], and as individual behavioural type adds phenotypic diversity to cannibalism propensity, individual strategies can have far-reaching consequences on higher-order processes. This conclusion should be viewed in light of the fact that the prey fishes in our experiments were large relative to cannibals [56] and that we have a small sample size. Holistic understanding of higher-order consequences should be pursued in light of size-structured cannibal-prey interactions and should be tested using a larger sample size. Nevertheless, assuming that our random sample of perch from the wild is representative of behavioural type frequencies across populations and fish sizes (but see [57] for limitations to this assumption), the link between behavioural type and cannibalism rates may have broader consequences. There were three times as many individuals of the asocial-inactive compared to the social-active behavioural type, and asocial-inactive individuals consumed significantly more conspecific prey than did individuals characterized as having a social-active phenotype. Although the skewed occurrence of behavioural types inevitably made the analyses unbalanced, we believe this result indicates that behavioural type can affect cannibalism rates in perch populations and may apply to other cannibalistic species with behavioural type diversity.

Although the precise mechanisms underpinning our results deserve attention in future work, we introduce the link between behavioural types and cannibalism propensity to shed light on the importance of bridging the order scales between individual behaviours and community processes.
